# Influences of temperature and humidity on cardiovascular disease among adults 65 years and older in China

**DOI:** 10.3389/fpubh.2022.1079722

**Published:** 2023-01-09

**Authors:** Huashuai Chen, Xuebin Zhang

**Affiliations:** ^1^Department of International Trade, Business School of Xiangtan University, Xiangtan, China; ^2^School of Urban and Regional Science, Shanghai University of Finance and Economics, Shanghai, China

**Keywords:** temperature, relative humidity, extreme temperature, CVD, older adults, China

## Abstract

**Background:**

The burden of cardiovascular disease (CVD) on the current aging society in China is substantial. Climate change, including extreme temperatures and humidity, has a detrimental influence on health. However, epidemiological studies have been unable to fully identify the association between climate change and CVD among older adults. Therefore, we investigated the associations between temperature and relative humidity and CVD among older adults in China.

**Methods:**

We used cohort data from the China Longitudinal Health and Longevity Survey (CLHLS) conducted in 2002, 2005, 2008, 2011, 2014, and 2018. A total of 39,278 Chinese adults 65 years and older participated in the analyses. The average annual temperatures and relative humidity during 2001 and 2017 (before the survey year) at the city level in China were used as the exposure measures. We selected patients with hypertension, heart disease, and stroke to create a sample of CVD patients. The associations between temperature and relative humidity and CVD were analyzed using the generalized estimation equation (GEE) model. Covariates included sociodemographic factors, health status, lifestyle, and cognitive function.

**Results:**

The average annual temperature was negatively correlated with the prevalence of CVD. Every 1°C increase in the average annual temperature reduced the rates of hypertension by 3% [odds ratio (OR): 0.97; 95% confidence interval (CI): 0.96–0.97], heart disease by 6% (OR: 0.94; 95% CI: 0.92–0.95), and stroke by 5% (OR: 0.95; 95% CI: 0.94–0.97). The results of the analyses stratified by sex, urban/rural residence, and educational level were robust. The average annual relative humidity was inversely associated with the likelihood of CVD among older adults. Every 1% increase in the average annual relative humidity reduced the rates of hypertension by 0.4% (OR: 0.996; 95% CI: 0.99–1.00), heart disease by 0.6% (OR: 0.994; 95% CI: 0.99–1.00), and stroke by 0.08% (OR: 0.992; 95% CI: 0.98–1.00). However, the effects were more obvious with higher humidity levels (>70).

**Conclusion:**

Our findings suggest that higher temperatures and relative humidity may reduce the risk of CVD among older adults.

## Introduction

Cardiovascular disease (CVD) is the leading cause of death and poses a critical public health challenge worldwide (1, 2). Since the mid-1970s, the rate of death attributable to CVD has markedly decreased in several high-income countries because of reductions in risk factors and improved countermeasures for CVD (3). In contrast, the incidence of CVD has been increasing in some low- and middle-income countries, with an estimated 80% of the global burden occurring in these countries (4). From 1990 to 2019, CVD was ranked first among the various causes of death in China. The Annual Report on Cardiovascular Health and Disease in China (2021) showed that CVD in rural and urban areas accounted for 46.74 and 44.26% of deaths, respectively, in 2019, and that two of every five deaths were caused by CVD (5). The current estimated number of CVD cases is 330 million. CVD is associated with high mortality and disability rates, which have inflicted heavy economic and social burdens on families and society.

In the context of steep climatic shifts, environmental risk factors have emerged as a major public health concern; therefore, an increasing number of studies have investigated the effects of temperature and humidity on CVD. CVD is highly sensitive to the weather of various environments worldwide (6). Regarding the association between temperature and CVD, studies have shown that temperature and CVD outcomes exist in a J, U, or V shape (7, 8). Both cold and heat deviating from the optimal temperature tend to increase the risk of CVD (7, 9–11). Specifically, according to previous studies, the significant inverse relationship between temperature and acute coronary syndrome (ACS) suggests that cold may have an important role in the incidence of ACS (12). The rates of death and hospital admissions attributable to CVD are higher in cold environments (13–15). However, scholars have emphasized that heat, especially extreme heat, is significantly correlated with the rate of death attributable to CVD (16–18).

The role of humidity, which is another important environmental factor, in human health outcomes has attracted increasing attention as well (19, 20). Some studies have implied that humidity is positively associated with health risks (21–23). High humidity could reduce the efficiency of the body to transport metabolic heat (11) and modify the effects of air pollution on health (24). However, the relationship between atmospheric humidity variations and CVD has not yet been confirmed. Although a few studies have found that humidity increases CVD and respiratory disease cases (25), a significant correlation between humidity and CVD has not been verified (14, 26, 27).

Extreme temperature events occur frequently with climate warming. China is the largest developing country in the world; therefore, its climate changes and environmental pollution may become more serious. Additionally, older adults are at relatively high risk for CVD (28), and the aging population in China is increasing. It is predicted that the risks of climate change associated with CVD will increase in the future. Therefore, it is of paramount importance to understand how long-term changes in temperature and humidity affect health. Furthermore, it is important to understand the adaptation mechanisms that may lead to mitigation of these effects. This study explored the associations between temperature and relative humidity and CVD among a nationally representative sample of older adults in China.

## Methods

### Study population

This research used data from the 2002–2018 waves of the Chinese Longitudinal Healthy Longevity Survey (CLHLS). The CLHLS is a nationwide survey conducted in 631 randomly selected cities and counties in 22 provinces, covering about 85% of the total population of China. More than 8,500 participants aged 80+ were interviewed at the 1998 baseline survey, and re-interviewed face-to-face every 2 or 3 years. Overall response rate is about 90% at each wave. In each follow-up survey, the CLHLS study re-interviewed older adults who participated in the previous wave and additionally included new recruitments to replace deceased and lost-to-follow-up respondents with the same sex and age. A complete description of the CLHLS is given elsewhere (29). The Peking University Ethics Committee accepted the research (IRB00001052–13074) after receiving informed permission from all participants and/or their family.

**Figure 1:**
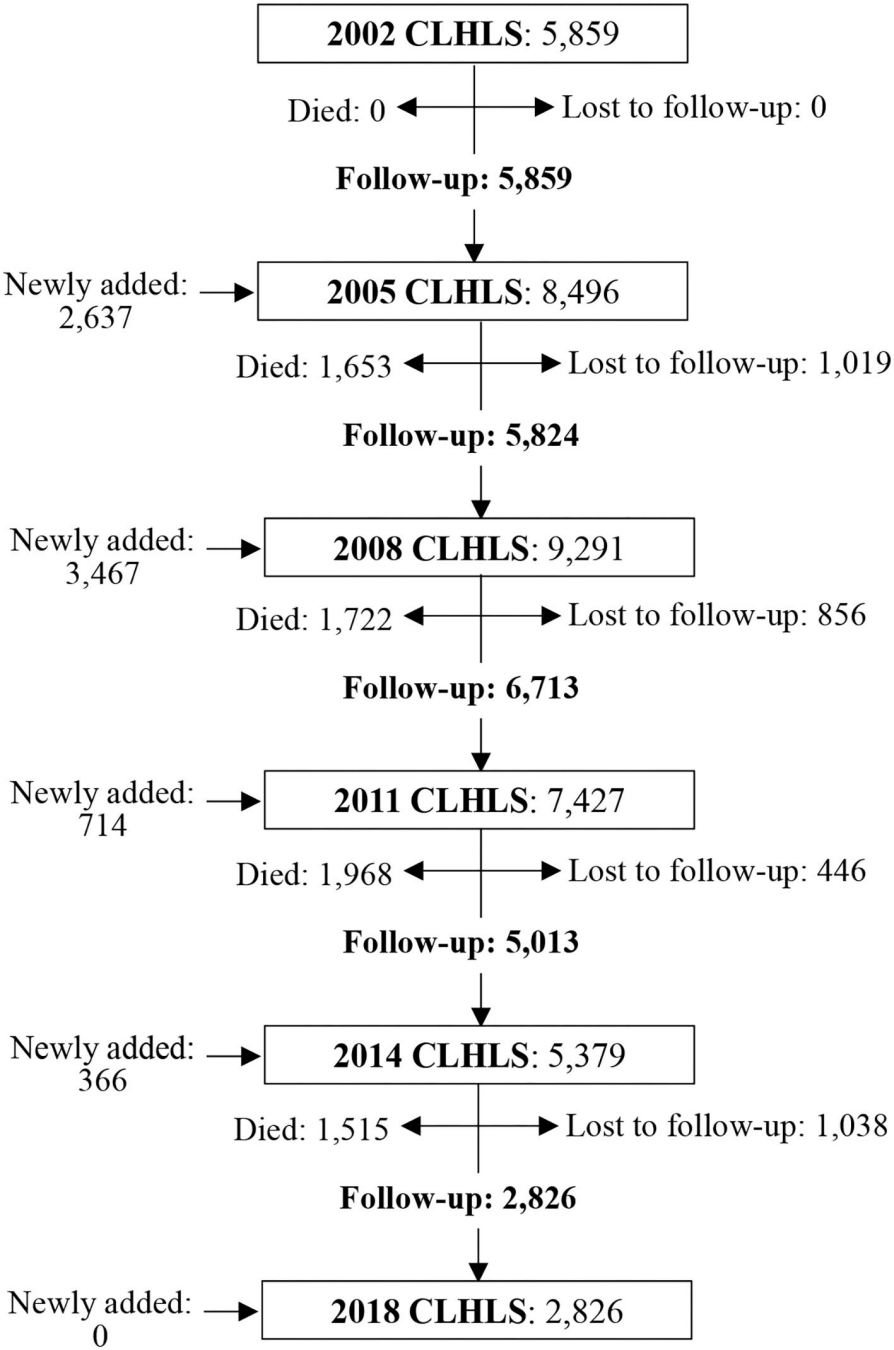
Flow chart of sample size across 6 waves of the CLHLS from 2002 to 2018. All the samples kept in the study were surveyed for at least two waves. Those samples who were surveyed only in one wave were removed.

Since 2002, the CLHLS adopted a wider sample design by interviewing approximately three randomly selected nearby elders aged 65–79 of predefined age and sex in conjunction with every two centenarians. Thus, we used data collected in waves 2002, 2005, 2008, 2011, 2014, and 2018, a total of 16 years. After excluding those samples that only participated in the survey for one wave, the final analysis comprised 39,728 older individuals. The flowchart illustrated data cleansing and inclusion and exclusion criteria (Figure 1).

### Assessment in residential temperature and relative humidity

Hourly city-level relative humidity (%) and temperature (°C) data in 2002, 2005, 2008, 2011, 2014, and 2018 were obtained from China Meteorological Data Network (http://data.cma.cn/). To evaluate temperature and relative humidity exposure for each CHLHS participant, hourly data was averaged yearly or seasonally using administrative codes corresponding to the cities where CLHLS samples were taken (non-publicly available information). For those cities without meteorological monitoring points, the missing values of temperature or humidity would be filled in by that of the most adjacent cities. The temperature and humidity exposures for this investigation were: (1) average relative humidity in the past year; (2) average temperature in the past year; (3) average relative humidity during the period from 1990 to the year of survey; (4) average temperature during the period from 1990 to the year of survey. To avoid seasonal bias, we additionally assessed the participants' exposure to temperature using the following: (5) average temperature during cold months; (6) average temperature during warm months. As in earlier publications, the warm months were defined as May through October, and the average temperature for these 6 months was defined as the average temperature for these months (30). January through April and November through December are the coldest months, and the average temperature for these 6 months is identical to the average temperature for each month. Extreme temperatures were depicted by the following: (7) daily maximum temperature for extreme heat; (8) daily lowest temperature for extreme cold. According to earlier publications (31, 32), and while attempting similar sample numbers among the grades similar and a rounded numerical critical point, the relative humidity average for the preceding year was divided into five categories: very low (<60), low (60–69.99), middle (70–74.99), high (75–79.99), and very high (≥80). The average annual temperature was divided into five categories: very low (<5), low (5–7.99), middle (8–9.99), high (10–12.99), and very high (≥13). As categorization factors, the average temperature during the warm months of the preceding year was separated into five categories: very low (<21), low (21–22.99), middle (23–24.49), high (24.5–25.99), and very high (≥26).

### CVD

CVD includes hypertension, myocardial infarction, stroke, heart failure, and other diseases involving the heart or blood vessels, which are mainly caused by microcirculation disorders. We assessed three primary composite outcome of major CVD events to identify whether the older adults suffer from CVD: (1) Having hypertension or not (yes = 1, no = 0). An older adult is viewed as “Having hypertension” if his/her systolic ≥ 140 or diastolic ≥ 90 in the physical examine of the survey. A person without hypertension is defined if his/her systolic < 140 and diastolic < 90. (2) Having heart disease or not (yes = 1, no = 0). (3) Having Stroke or cerebrovascular disease or not (yes = 1, no = 0). Whether the participant had the last two diseases was self-reports based on the medical record or the doctor's diagnosis. The samples would be removed if they reported to have heart disease or Stroke/cerebrovascular disease but have neither medical record nor the doctor's diagnosis.

### Covariates

To limit the influence of possible confounders, we analyzed current research from PubMed published within the previous decade to identify factors as covariates, including frequent CVD predictors. Sex (male or female), age, geographical location (east, central, or western), residence (urban or rural), marital status [single (i.e., divorced, widowed, never married) or married], education (illiterate, fewer than 6 years of education, or at least 6 years of education), and economic status [logarithm of per capita income = ln(per capita household income + 1)] comprised the variables. Per capita household income in CNY denotes the total annual household income divided by the number of family members. A restriction in any activity of daily life, including bathing, dressing, using the restroom, indoor transferring, continence, or eating, is a handicap. Cognitive functioning was assessed based on the Chinese version of the Mini Mental State Examination (MMSE), one of the most widely used instruments for measuring cognitive health in older persons and recording cognitive shifts (33, 34).

### Statistical analyses

This research studied the impact of temperature and relative humidity on the incidence of CVD. The participants' baseline attributes were summarized according to CVD existence or otherwise. We reported data for continuous variables as means and standard deviations (SD), and for categorical variables as frequencies and percentages. The generalized estimating equation (GEE) was used to examine the associations between temperature, relative humidity, and CVD. By examining the data correlational structure, GEE allows us to develop efficient and objective regression parameters (35). Based on the model's estimated robust standard errors, the Logit link function was used to construct odds ratios (ORs) and 95% confidence intervals (CIs). To accommodate for repeated measurements at the subject level, we applied an exchangeable correlation structure. In the models, potential confounding variables such as demographic, health, and psychological aspects had been accounted for. Statistical treatment was conducted using the Stata 16 statistical program.

## Results

### Description of the study sample

This study included a total of 39,278 Chinese older adult observations. The mean age of the older adults in the sample was 83.99 ± 11.0 years; the gender composition was generally balanced (46.5% men). The prevalence of CVD was 58.13%, the proportions of hypertension, heart disease and stroke in the total sample are 51.3, 12.1, and 7.6% respectively. Approximately one-quarter (24.5%) of the older adults lived in urban areas. The geographical distribution of samples was relatively balanced, nearly half (46%) lived in east China. In general, the education of the Chinese older adult is low, 53% of the participants were illiterate. The marriage samples accounted for 40.6%. The average number of surviving children of the older adults is 3.54 ± 1.88. The logarithm of the average income of the older adults is 8.21 ± 1.47. 20% of the older adults suffered from disability in activities of daily living, and 16.3% of them had cognitive impairment (Table 1).

**Table 1:** Characteristics of the study samples, CLHLS 2002–2018.

	**All samples**	**No hypertension**	**Having hypertension**	***p*-Values of difference**
	***N* = 39,278**	***N*= 19,129**	***N* = 20,149**	
Having hypertension, *n* (%)	20,149 (51.3)	0 (0.0)	20,149 (100.0)	
Having heart disease, *n* (%)	4,545 (12.1)	1,813 (9.9)	2,732 (14.2)	<0.001
Having stroke or cerebrovascular disease, *n* (%)	2,848 (7.6)	1,139 (6.2)	1,709 (8.8)	<0.001
**Temperature and humidity**				
Average relative humidity in the past year, mean (SD)	71.44 (7.76)	71.88 (7.67)	71.02 (7.84)	<0.001
**Grades**				
Very low: < 60, *n* (%)	4,006 (10.2)	1,771 (9.3)	2,235 (11.1)	< 0.001
Low: 60–69.99, *n* (%)	11,384 (29.0)	5,229 (27.3)	6,155 (30.5)	<0.001
Middle: 70–74.99, *n* (%)	9,462 (24.1)	4,636 (24.2)	4,826 (24.0)	0.511
High: 75–79.99, *n* (%)	9,548 (24.3)	5,000 (26.1)	4,548 (22.6)	<0.001
Very high: ≥80, *n* (%)	4,878 (12.4)	2,493 (13.0)	2,385 (11.8)	<0.001
Average temperature in the past year, mean (SD)	16.20 (3.95)	16.47 (3.87)	15.94 (4.01)	<0.001
**Grades**				
Very low: <12°C, *n* (%)	4,016 (10.2)	1,667 (8.7)	2,349 (11.7)	<0.001
Low: 12–14.99°C, *n* (%)	8,153 (20.8)	3,697 (19.3)	4,456 (22.1)	<0.001
Middle: 15–16.99°C, *n* (%)	10,416 (26.5)	5,106 (26.7)	5,310 (26.4)	0.447
High: 17–19.99°C, *n* (%)	10,993 (28.0)	5,503 (28.8)	5,490 (27.2)	<0.001
Very high: ≥20°C, *n* (%)	5,700 (14.5)	3,156 (16.5)	2,544 (12.6)	<0.001
Average temperature in the cold months of past year, mean (SD)	8.71 (5.58)	9.10 (5.46)	8.34 (5.66)	<0.001
**Grades**				
Very low: < 5°C, *n* (%)	6,973 (17.8)	2,925 (15.3)	4,048 (20.1)	<0.001
Low: 5–7.99°C, *n* (%)	8,964 (22.8)	4,285 (22.4)	4,679 (23.2)	0.053
Middle: 8–9.99°C, *n* (%)	7,566 (19.3)	3,661 (19.1)	3,905 (19.4)	0.543
High: 10–12.99°C, *n* (%)	8,924 (22.7)	4,562 (23.8)	4,362 (21.6)	<0.001
Very high: ≥13°C, *n* (%)	6,851 (17.4)	3,696 (19.3)	3,155 (15.7)	<0.001
Average temperature in the warm months of past year, mean (SD)	23.57 (2.46)	23.73 (2.42)	23.42 (2.48)	<0.001
**Grades**				
Very low: < 21°C, *n* (%)	4,445 (11.3)	1,874 (9.8)	2,571 (12.8)	<0.001
Low: 21–22.99°C, *n* (%)	9,591 (24.4)	4,555 (23.8)	5,036 (25.0)	0.006
Middle: 23–24.49°C, *n* (%)	11,694 (29.8)	5,728 (29.9)	5,966 (29.6)	0.469
High: 24.5–25.99°C, *n* (%)	7,726 (19.7)	3,759 (19.7)	3,967 (19.7)	0.926
Very high: ≥26°C, *n* (%)	5,822 (14.8)	3,213 (16.8)	2,609 (12.9)	<0.001
Average of daily maximum temperature in past year, mean (SD)	20.98 (3.64)	21.23 (3.57)	20.75 (3.70)	<0.001
Average of daily minimum temperature in past year, mean (SD)	12.53 (4.57)	12.84 (4.49)	12.23 (4.63)	<0.001
Average temperature during the period from 1990 to the year of survey	16.11 (3.98)	16.40 (3.90)	15.83 (4.04)	<0.001
Average relative humidity during the period from 1990 to the year of survey	73.64 (7.02)	74.10 (6.89)	73.22 (7.12)	<0.001
**Covariates**				
Living in urban area, *n* (%)	9,631 (24.5)	4,778 (25.0)	4,853 (24.1)	0.040
East China, *n* (%)	18,084 (46.0)	8,214 (42.9)	9,870 (49.0)	<0.001
Male, *n* (%)	18,245 (46.5)	8,959 (46.8)	9,286 (46.1)	0.137
Age, mean (SD)	83.99 (11.00)	84.04 (11.29)	83.94 (10.71)	0.347
**Age group**				
65–79, *n* (%)	14,977 (38.9)	7,416 (39.6)	7,561 (38.1)	0.003
80–89, *n* (%)	11,360 (29.5)	5,204 (27.8)	6,156 (31.1)	<0.001
90–99, *n* (%)	8,686 (22.5)	4,272 (22.8)	4,414 (22.3)	0.195
100+, *n* (%)	3,523 (9.1)	1,830 (9.8)	1,693 (8.5)	<0.001
**Education**				
Illiterates, *n* (%)	20,809 (53.0)	10,195 (53.3)	10,614 (52.7)	0.220
Elementary school, *n* (%)	12,076 (30.7)	5,960 (31.2)	6,116 (30.4)	0.085
Middle school or higher, *n* (%)	6,393 (16.3)	2,974 (15.5)	3,419 (17.0)	<0.001
Current married, *n* (%)	15,932 (40.6)	7,837 (41.0)	8,095 (40.2)	0.109
# of alive children, mean (SD)	3.54 (1.88)	3.52 (1.87)	3.55 (1.89)	0.200
Log of income per capita, mean (SD)	8.21 (1.47)	8.19 (1.47)	8.24 (1.48)	<0.001
ADL disabled, *n* (%)	7,840 (20.0)	3,826 (20.0)	4,014 (19.9)	0.850
Cognitive impairment, *n* (%)	6,171 (16.3)	3,020 (16.5)	3,151 (16.2)	0.495

All the samples kept in the study were surveyed for at least two waves. Those samples who were surveyed only in one wave were removed.

### Temperature and relative humidity exposure

Table 1 also summarized the average temperature and average relative humidity and to which the older adults was exposed. The average temperature during the cold months and warm months of the previous year was 8.98 ± 5.58 and 23.70 ± 2.42°C, respectively. Over the past year, the average daily maximum temperature and minimum temperature was 21.17 ± 3.63 and 12.73 ± 4.54°C, respectively. The average temperature in the past year was 16.40 ± 3.93°C, while the average relative humidity (mean ± SD) in the past year was 71.88 ± 7.99%. Average temperature and average relative humidity during the period from 1990 to the year of survey were 16.11 ± 3.98°C and 73.64 ± 7.02%, respectively.

### Associations of temperature and relative humidity with CVD

The average temperature over the past year was negatively correlated with the prevalence of CVD. In the past year, every 1°C increase in temperature would reduce the prevalence of hypertension by 3% (OR: 0.97; 95% CI: 0.96–0.97), heart disease by 6% (OR: 0.94; 95% CI: 0.92–0.95), and stroke by 5% (OR: 0.95; 95% CI: 0.94–0.97). Every 1% increase in average relative humidity in the past year would reduce the prevalence of hypertension by 0.4% (OR: 0.996; 95% CI: 0.99–1.00), heart disease by 0.6% (OR: 0.994; 95% CI: 0.99–1.00), and stroke by 0.08% (OR: 0.992; 95% CI: 0.98–1.00; Table 2). Based on the subgroup analyses, we found that the associations of temperature and CVD were robust across subgroups of other risk factors, including sex, urban/rural residence, and level of educational attainment. However, the effect of humidity on different CVD varied among subgroups. We found that the increase of relatively humidity would reduce the prevalence of hypertension and heart disease among female samples, hypertension and stroke among rural residents, heart disease and stroke among illiterate samples (Table 3).

**Table 2:** Effects of temperature and relative humidity on CVD among Chinese older adults aged 65+ during 2002–2018: Odds Ratios (OR) from GEE models.

**Dependent variables**	Having hypertension (No^*^)	Having heart disease (No^*^)	Having stroke (No^*^)	Having hypertension (No^*^)	Having heart disease (No^*^)	Having stroke (No^*^)
	**OR [95% CI]**	**OR [95% CI]**	**OR [95% CI]**	**OR [95% CI]**	**OR [95% CI]**	**OR [95% CI]**
Average temperature in the past year	0.97 [0.96, 0.97]^***^	0.94 [0.92, 0.95]^***^	0.95 [0.94, 0.97]^***^			
Average relative humidity in the past year	0.996 [0.99, 1.00]^*^	0.994 [0.99, 1.00]^*^	0.992 [0.98, 1.00]^*^			
Average temperature during the period from 1990 to the year of survey				0.96 [0.95, 0.97]^***^	0.93 [0.91, 0.94]^***^	0.96 [0.94, 0.97]^***^
Average relative humidity during the period from 1990 to the year of survey				1.000 [0.99, 1.005]	1.003 [0.99, 1.012]	0.992 [0.98, 1.002]
**Covariates**						
Urban residence (urban^*^)	0.88 [0.83, 0.93]^***^	2.03 [1.86, 2.22]^***^	1.61 [1.43, 1.80]^***^	0.88 [0.83, 0.93]^***^	2.04 [1.87, 2.23]^***^	1.60 [1.43, 1.79]^***^
East provinces (middle/west^*^)	1.26 [1.20, 1.32]^***^	1.16 [1.07, 1.27]^***^	0.95 [0.86, 1.06]	1.27 [1.21, 1.33]^***^	1.20 [1.09, 1.31]^***^	0.95 [0.86, 1.06]
Male (female^*^)	0.94 [0.89, 0.99]^**^	0.66 [0.60, 0.73]^***^	1.27 [1.14, 1.41]^***^	0.94 [0.89, 0.99]^**^	0.67 [0.60, 0.73]^***^	1.27 [1.14, 1.41]^***^
**Age groups (65**~79 years old^*^)						
80~89 years old	1.12 [1.06, 1.18]^***^	0.93 [0.85, 1.01]^*^	0.72 [0.64, 0.80]^***^	1.12 [1.06, 1.18]^***^	0.92 [0.85, 1.01]^*^	0.72 [0.64, 0.80]^***^
90~99 years old	0.99 [0.93, 1.06]	0.68 [0.60, 0.76]^***^	0.40 [0.35, 0.47]^***^	0.99 [0.93, 1.06]	0.68 [0.60, 0.76]^***^	0.40 [0.35, 0.47]^***^
100+ years old	0.89 [0.81, 0.98]^**^	0.48 [0.40, 0.58]^***^	0.18 [0.14, 0.23]^***^	0.89 [0.81, 0.98]^**^	0.48 [0.40, 0.58]^***^	0.18 [0.14, 0.23]^***^
**Years of schooling (0 year** ^*^ **):**						
1~6 years	1.02 [0.96, 1.08]	1.22 [1.10, 1.35]^***^	0.98 [0.87, 1.11]	1.02 [0.96, 1.08]	1.22 [1.10, 1.35]^***^	0.98 [0.87, 1.11]
7+ years	1.07 [0.99, 1.15]^*^	1.51 [1.34, 1.71]^***^	0.96 [0.83, 1.12]	1.07 [0.99, 1.15]^*^	1.51 [1.34, 1.71]^***^	0.96 [0.83, 1.12]
Current married (unmarried^*^)	0.92 [0.87, 0.97]^***^	1.10 [1.000, 1.20]^**^	1.12 [1.001, 1.24]^**^	0.92 [0.87, 0.97]^***^	1.10 [1.001, 1.20]^**^	1.12 [1.001, 1.24]^**^
# of alive children	1.01 [1.001, 1.03]^**^	1.03 [1.01, 1.05]^***^	1.03 [1.004, 1.06]^**^	1.01 [1.001, 1.03]^**^	1.03 [1.01, 1.05]^***^	1.03 [1.004, 1.06]^**^
Log of income per capita	1.004 [0.99, 1.02]	1.01 [0.99, 1.04]	1.02 [0.99, 1.05]	1.003 [0.99, 1.02]	1.01 [0.99, 1.04]	1.02 [0.99, 1.05]
ADL disabled (active^*^)	0.96 [0.90, 1.02]	1.57 [1.43, 1.72]^***^	3.50 [3.12, 3.92]^***^	0.96 [0.90, 1.02]	1.57 [1.44, 1.72]^***^	3.50 [3.13, 3.93]^***^
Cognitive impairment (active^*^)	1.01 [0.95, 1.08]	0.84 [0.76, 0.93]^***^	1.40 [1.24, 1.58]^***^	1.01 [0.95, 1.08]	0.84 [0.76, 0.93]^***^	1.40 [1.24, 1.58]^***^
**Wave (2002** ^*^ **)**						
2005	0.74 [0.69, 0.79]^***^	1.23 [1.11, 1.36]^***^	1.35 [1.17, 1.57]^***^	0.76 [0.71, 0.81]^***^	1.30 [1.17, 1.44]^***^	1.41 [1.22, 1.63]^***^
2008	0.96 [0.89, 1.03]	1.33 [1.19, 1.48]^***^	1.54 [1.32, 1.79]^***^	0.99 [0.92, 1.06]	1.39 [1.25, 1.56]^***^	1.58 [1.36, 1.84]^***^
2011	0.90 [0.84, 0.97]^***^	1.81 [1.61, 2.04]^***^	1.87 [1.60, 2.19]^***^	0.95 [0.88, 1.02]	1.98 [1.76, 2.23]^***^	1.99 [1.70, 2.32]^***^
2014	1.08 [0.999, 1.18]^*^	2.19 [1.93, 2.49]^***^	2.24 [1.89, 2.65]^***^	1.10 [1.02, 1.20]^**^	2.26 [1.99, 2.57]^***^	2.25 [1.90, 2.66]^***^
2018	1.19 [1.07, 1.33]^***^	2.41 [2.05, 2.84]^***^	3.43 [2.78, 4.24]^***^	1.20 [1.08, 1.34]^***^	2.47 [2.09, 2.90]^***^	3.41 [2.76, 4.21]^***^

* p < 0.10.

** p < 0.05.

*** p < 0.01.

OR, odds ratio; CI, confidence interval.

Generalized estimation equation (GEE) were used.

**Table 3:** Effects of temperature and relative humidity and on CVD during 2002–2018, by subgroups models.

**Dependent variables**	Having hypertension (No^*^)	Having heart disease (No^*^)	Having stroke (No^*^)
	**OR [95% CI]**	**OR [95% CI]**	**OR [95% CI]**
**Model 1: Males only**			
Average temperature in the past year	0.97 [0.96, 0.98]^***^	0.94 [0.92, 0.96]^***^	0.95 [0.93, 0.97]^***^
Average relative humidity in the past year	0.998 [0.99, 1.004]	1.000 [0.99, 1.01]	0.991 [0.98, 1.002]
Covariates	√	√	√
**Model 2: Females only**			
Average temperature in the past year	0.97 [0.96, 0.98]^***^	0.93 [0.92, 0.95]^***^	0.96 [0.94, 0.98]^***^
Average relative humidity in the past year	0.994 [0.99, 1.00]^*^	0.99 [0.98, 0.998]^**^	0.993 [0.98, 1.004]
Covariates	√	√	√
**Model 3: Urban residents**			
Average temperature in the past year	0.97 [0.96, 0.98]^***^	0.95 [0.93, 0.97]^***^	0.97 [0.95, 0.99]^**^
Average relative humidity in the past year	0.995 [0.99, 1.002]	0.996 [0.99, 1.01]	0.998 [0.99, 1.01]
Covariates	√	√	√
**Model 4: Rural residents**			
Average temperature in the past year	0.97 [0.96, 0.98]^***^	0.92 [0.90, 0.93]^***^	0.94 [0.92, 0.96]^***^
Average relative humidity in the past year	0.994 [0.99, 1.00]^**^	0.998 [0.99, 1.01]	0.989 [0.98, 1.00]^*^
Covariates	√	√	√
**Model 5: 0 year schooling samples**			
Average temperature in the past year	0.96 [0.95, 0.98]^***^	0.92 [0.91, 0.94]^***^	0.95 [0.93, 0.97]^***^
Average relative humidity in the past year	0.997 [0.99, 1.002]	0.990 [0.98, 1.00]^*^	0.99 [0.97, 0.997]^**^
Covariates	√	√	√
**Model 6: 1**+ **year schooling samples**			
Average temperature in the past year	0.97 [0.96, 0.98]^***^	0.95 [0.93, 0.96]^***^	0.96 [0.94, 0.98]^***^
Average relative humidity in the past year	0.995 [0.99, 1.00]^*^	0.997 [0.99, 1.01]	0.996 [0.99, 1.01]
Covariates	√	√	√

* p < 0.10.

** p < 0.05.

*** p < 0.01.

OR, odds ratio; CI, confidence interval.

Generalized estimation equation (GEE) were used. All samples are included. Covariates are the same as in Table 2, the Odds ratios of which are not listed.

We conducted sensitivity analyses through the following methods to check the robustness of our conclusions: using the average temperature and relative humidity during the period from 1990 to the year of survey (Table 1, Columns 4–5), the average temperature in the warm months (Model 1), the average temperature in the cold months (Model 2), the average daily maximum temperature (Model 3), and the average daily minimum temperature (Model 4) as alternative indicators to regress. The estimation result of the influence of temperature on CVD was consistent with our main findings. Notably, the effect of humidity on CVD disease started to highlight only when we used “average temperature in the warm months of past year” and “average of daily maximum temperature in the past year” as the temperature indicator in Model 2, which indicated that temperature and humidity may have interactive effects on CVD (Table 4).

**Table 4:** Effects of temperature and relative humidity on CVD during 2002–2018: Odds ratios (OR) from hierarchical GEE models.

**Dependent variables**	Having hypertension (No*)	Having heart disease (No*)	Having stroke (No*)
	**OR [95%CI]**	**OR [95%CI]**	**OR [95%CI]**
**Model 1:**			
Average temperature in the cold months of past year	0.97 [0.97, 0.98]^***^	0.95 [0.94, 0.96]^***^	0.97 [0.96, 0.98]^***^
Average relative humidity in the past year	0.997 [0.99, 1.001]	0.996 [0.99, 1.003]	0.994 [0.99, 1.002]
Covariates	√	√	√
**Model 2:**			
Average temperature in the warm months of past year	0.95 [0.94, 0.96]^***^	0.92 [0.90, 0.94]^***^	0.94 [0.92, 0.96]^***^
Average relative humidity in the past year	0.993 [0.99, 0.997]^***^	0.989 [0.98, 0.99]^***^	0.989 [0.98, 0.996]^***^
Covariates	√	√	√
**Model 3:**			
Average of daily maximum temperature in the past year	0.96 [0.96, 0.97]^***^	0.93 [0.92, 0.94]^***^	0.95 [0.94, 0.97]^***^
Average relative humidity in the past year	0.995 [0.99, 0.999]^**^	0.993 [0.99, 0.999]^**^	0.991 [0.98, 0.998]^**^
Covariates	√	√	√
**Model 4:**			
Average of daily minimum temperature in the past year	0.97 [0.96, 0.98]^***^	0.94 [0.93, 0.96]^***^	0.96 [0.95, 0.97]^***^
Average relative humidity in the past year	0.996 [0.99, 1.001]	0.996 [0.99, 1.002]	0.994 [0.99, 1.002]
Covariates	√	√	√

Note: The same as Table 3.

**Figure 2:**
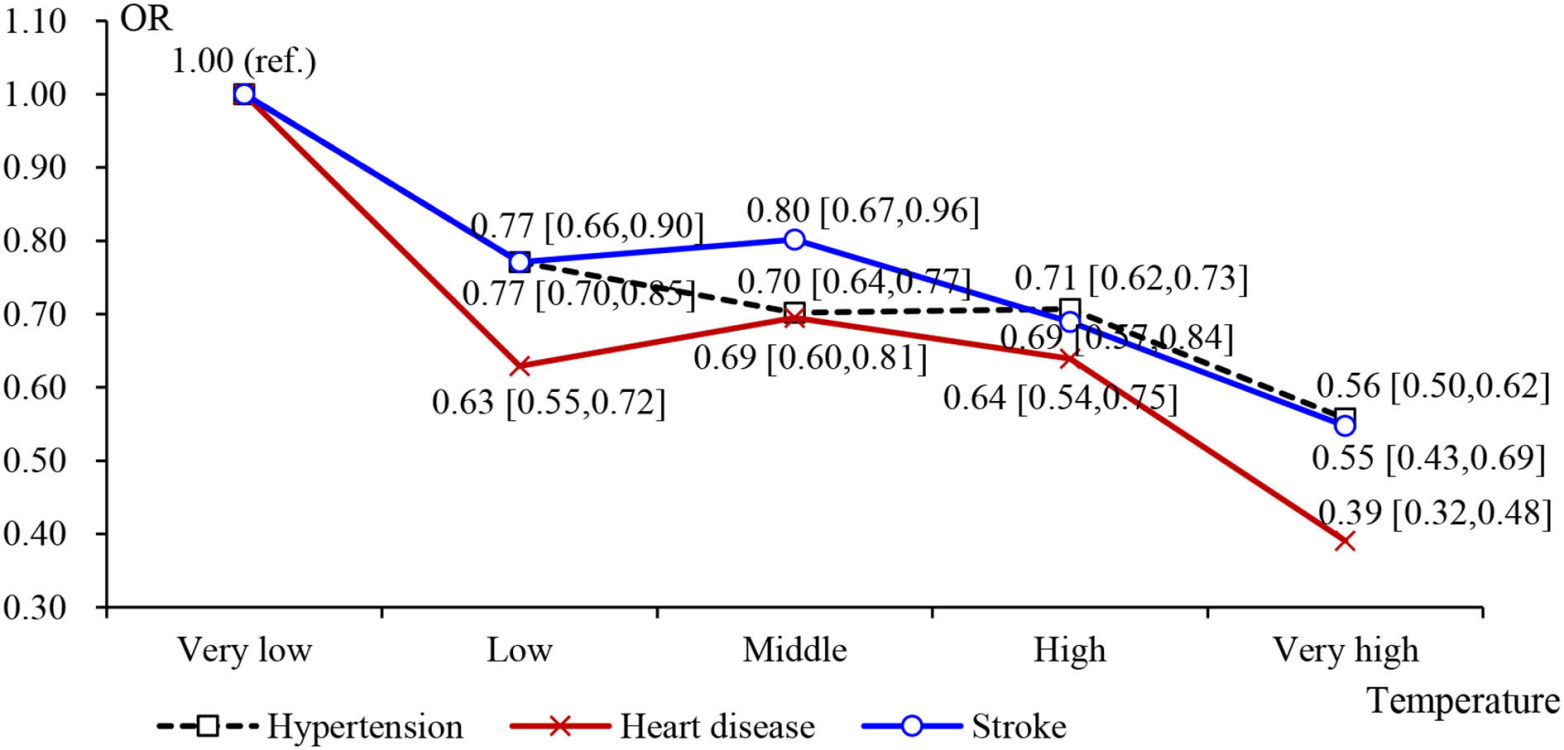
Effects of categorical average temperature on CVD: Odds ratios (OR) in Table 5.

**Figure 3:**
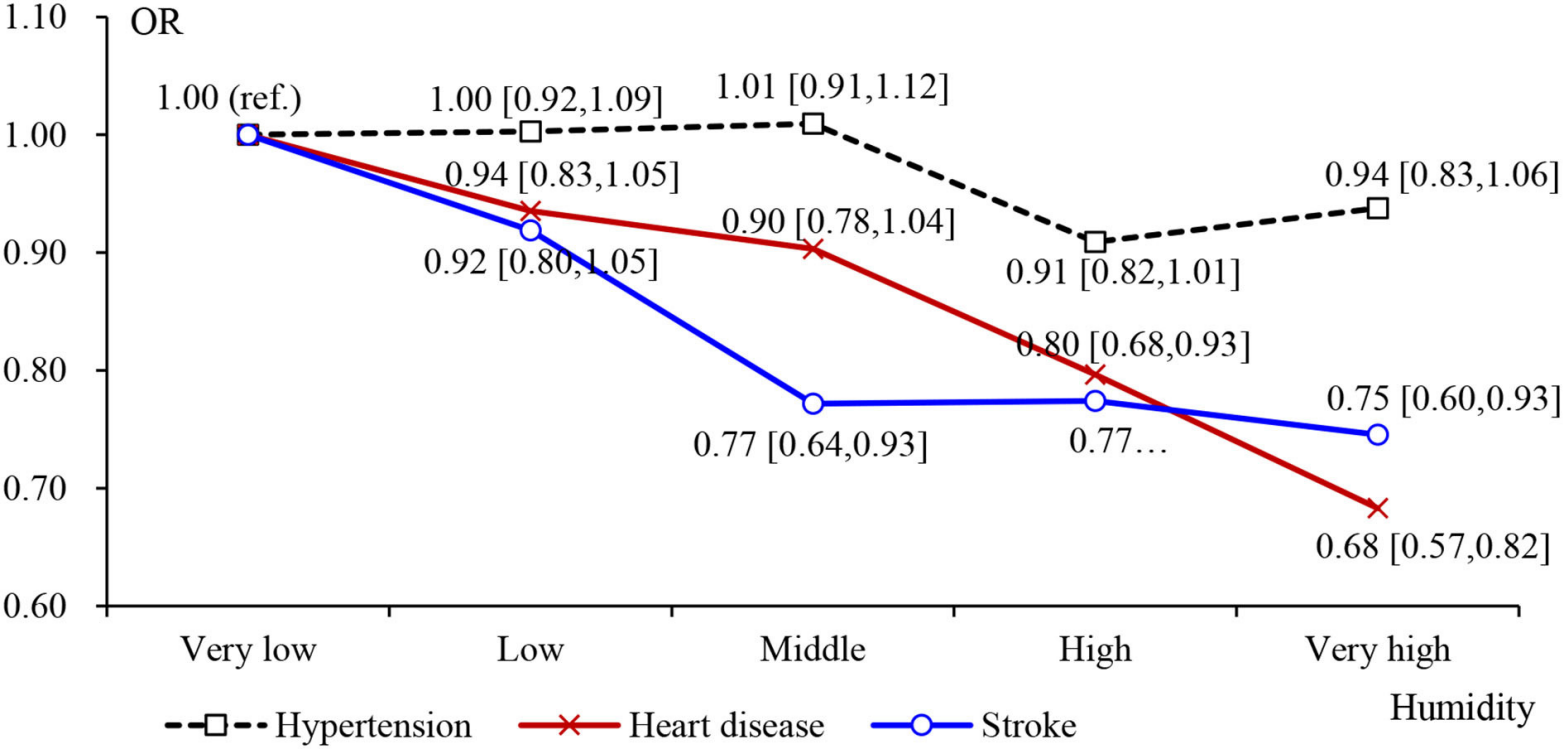
Effects of categorical average humidity on CVD: Odds ratios (OR) in Table 5.

Furthermore, considering the potential non-linear relationship between temperature and relative humidity and CVD, we modeled temperature and relative temperature from “very low” to “very high” as categorical variables, with “very low” as the reference group. According to Table 5 and Figure 2, we found a non-linear relationship between the temperature and CVD risk. It meant that the adverse effect of temperature on cardiovascular disease increased with the temperature getting higher. Similar results appeared in the impact of humidity on CVD. The difference was that the increase of humidity did not obviously reduce the prevalence of hypertension (Figure 3). In the “low” interval, humidity increased may lead to a high prevalence of CVD, but in the “middle” interval and above, humidity showed a significant negatively correlated with the prevalence of CVD.

**Table 5:** Effects of categorical temperature and relative humidity on CVD: Odds ratios (OR) from hierarchical GEE models.

**Dependent variables**	Having hypertension (No*)	Having heart disease (No*)	Having Stroke (No*)
	**OR [95% CI]**	**OR [95% CI]**	**OR [95% CI]**
**Average temperature in the past year**			
Very low: < 12°C (ref.)	1.00	1.00	1.00
Low: 12–14.99°C	0.77 [0.70, 0.85]^***^	0.63 [0.55, 0.72]^***^	0.77 [0.66, 0.90]^***^
Middle: 15–16.99°C	0.70 [0.64, 0.77]^***^	0.69 [0.60, 0.81]^***^	0.80 [0.67, 0.96]^**^
High: 17–19.99°C	0.71 [0.64, 0.78]^***^	0.64 [0.54, 0.75]^***^	0.69 [0.57, 0.84]^***^
Very high: ≥20°C	0.56 [0.50, 0.62]^***^	0.39 [0.32, 0.48]^***^	0.55 [0.43, 0.69]^***^
**Average relative humidity in the past year**			
Very low: < 60 (ref.)	1.00	1.00	1.00
Low: 60–69.99	1.00 [0.92, 1.09]	0.94 [0.83, 1.05]	0.92 [0.80, 1.05]
Middle: 70–74.99	1.01 [0.91, 1.12]	0.90 [0.78, 1.04]	0.77 [0.64, 0.93]^***^
High: 75–79.99	0.91 [0.82, 1.01]*	0.80 [0.68, 0.93]^***^	0.77 [0.64, 0.94]^***^
Very high: ≥80	0.94 [0.83, 1.06]	0.68 [0.57, 0.82]^***^	0.75 [0.60, 0.93]^***^
Covariates	√	√	√

Note: The same as Table 3.

## Discussion

Among the data collected by the CLHLS, we found significant associations between the annual average temperature and prevalence of CVD. The ORs of hypertension, heart disease, and stroke associated with temperature were 0.97 (95% CI: 0.96–0.97), 0.94 (95% CI: 0.92–0.95), and 0.95 (95% CI: 0.94–0.97), respectively. The negative relationship between relative humidity and CVD was significant at a level of 10%, as shown by some subgroup analyses. Furthermore, we found that the effects of temperature and humidity on CVD were non-linear. As the temperature increased, the impact on CVD increased. Additionally, the influence of humidity on CVD only occurs at high levels. These associations indicate that lower temperatures and humidity may be linked to a higher incidence of CVD among older individuals.

We found that exposure to lower temperatures and humidity was associated with an increased risk of CVD. In terms of the influence of temperature on CVD, recent reviews provided evidence that both cold and heat would increase mortality after deviating from the optimal temperature; however, the epidemiological studies were conducted in different areas where cold effects were stronger than heat effects (12, 36, 37). Similar findings were reported by previous studies conducted in China. Chen et al. (38) found that ~14% of deaths were related to ambient temperature; of those 14, 11% were related to cold temperatures. The reason for higher mortality attributable to CVD at low temperatures may be that the mortality risk at cold temperatures lasted more than 14 days; however, that at hot temperatures occurred immediately and lasted 2–3 days. In support of our findings, Yang et al. (39) found that 17.1% (95% CI: 14.4–19.1) of deaths attributable to CVD (330,352 deaths) were associated with ambient temperature. Most of these deaths (15.8%; 95% CI: 13.1–17.9) were attributable to cold. According to a study by Xu et al. (40), most deaths attributable to CVD were associated with moderately cold temperatures.

The mechanism by which temperature affects CVD remains undetermined; however, it may involve changes in vascular tone, the autonomic nervous system response, and oxidative stress (39). Saeki et al. (41) found that a 1°C lower indoor temperature was significantly associated with a 0.22-mm Hg higher daytime systolic blood pressure and 0.34-mm Hg higher sleep-trough morning blood pressure surge when repeated measurements were performed on two consecutive days during colder months (October–April) among 868 older adults in Japan, indicating that low temperatures would lead to changes in vascular tone. Hanna suggested that activation of the sympathetic nervous system and secretion of catecholamines increased in response to cold temperatures, which could result in increased blood pressure through increased heart rate and peripheral vascular resistance (42). Another study of rats also demonstrated changes in sympathetic neural activity when exposed to a cold room (4°C) (43). Oxidative stress may be a crucial mechanism by which temperature affects CVD. Luo et al. (44) showed that exposure to cold caused a significant increase in inflammatory cytokines and methane dicarboxylic aldehyde and a decrease in superoxide dismutase and glutathione peroxidase activity. Cold temperatures could also increase endothelial nitric oxide synthase expression, which leads to the development of hypertension and impaired endothelial vasodilator function in isolated arterial tissue (45).

Regarding humidity, as suggested by most related studies, the negative correlation between the average annual relative humidity and the risk of CVD in GEE models is generally not stable (14, 26). However, the results regarding the impact of the categorical average relatively humidity on CVD showed that the rates of heart disease and stroke were significantly reduced with moderate or higher humidity levels. Abrignani et al. (27) also verified the non-linear relationship between humidity and CVD and found a significant negative association between the incidence relative ratio (95% CIs/unit of measurement) and daily number of hospital admissions for angina and maximal humidity; however, this association was positive with minimal humidity. Humidity may have an impact on cardiac function because of its effects on high body core temperatures and low hydration levels, both of which might be associated with the atmospheric moisture state and increased cardiac function or work (46). Additionally, the effect of humidity on CVD varies among subgroups of individual characteristics; however, the effect of socioeconomic status on CVD was the same as that reported by previous studies (47, 48).

Compared to other studies of the effects of temperature and humidity on CVD in China, this study had two unique advantages. First, the samples included in previous studies were mostly limited to a single region or some large cities (39, 49, 50). The samples included in this study were from half of the counties and cities (randomly selected) in 23 of the 31 provinces in China. The regional differences in temperature and humidity experienced by the samples in our study were larger; therefore, the effects of temperature and humidity on CVD could be more clearly identified. Second, in most previous studies, time-series regression was used to analyze the outpatient data of hospitals without considering the individual characteristics of the samples (14, 50, 51). Some studies have controlled for a few variables, such as age, sex, and education; however, they failed to sufficiently consider the effects of economic status and lifestyles on the results (38, 39). To our knowledge, this is the first study to consider the economic status and lifestyles of older adults. Additionally, the intergenerational support received by older adults was further analyzed, and the interference of factors that may confound the effects of temperature and humidity on CVD, such as different living environments, different medical conditions, and the use of air conditioners, were excluded. Therefore, the accuracy of the results was confirmed. Because of the drastic climate changes across the globe and aging population in China, it is intriguing to study the relationships between temperature, relative humidity, and age-related conditions. We believe that our findings will increase the attention focused on cardiovascular health issues, such hypertension, heart disease, stroke, and other CVDs, among older adults in China and their associations with weather changes, thereby leading to improvements in their quality of life.

This study had several limitations. First, because of the lack of detailed biomedical indicators in the questionnaire data, we were unable to conduct a more in-depth analysis of the effects of temperature and humidity on CVD. Second, the impact of other environmental factors, in addition to temperature and humidity, on CVD should be explored because some may interfere with the effects of temperature and humidity on CVD. For example, northern China uses coal for heating in winter; therefore, cold temperatures are often accompanied by high pollution rates, which may have affected the estimation results of this study. Furthermore, personal exposure varies with indoor ventilation and indoor activity; therefore, it may be appropriate to measure indoor temperature and relative humidity.

In conclusion, we found that the average annual temperature was negatively associated with the CVD likelihood and the inhibitory effect of the annual moderate or higher relative humidity on the prevalence of heart disease and stroke among older adults. Our findings indicate that extreme cold and low humidity are independently associated with a higher likelihood of CVD among older adults.

## Data availability statement

Publicly available datasets were analyzed in this study. This data can be found at: https://agingcenter.duke.edu/CLHLS.

## Ethics statement

Ethical approval for the CLHLS was obtained from the Research Ethics Committees of Peking University and Duke University, and all participants provided written informed consent. All methods were performed in accordance with the relevant ethical guidelines and regulations. The patients/participants provided their written informed consent to participate in this study.

## Author contributions

HC contributed to perform the data analyses and the design and conception of the study. XZ contributed to write the manuscript. All authors contributed to the article and approved the submitted version.
